# In Memoriam: Paul Ekman (February 15, 1934 - November 17, 2025)

**DOI:** 10.1007/s42761-025-00346-5

**Published:** 2025-12-13

**Authors:** Robert W. Levenson

**Affiliations:** Department of Psychology, University of California, Berkeley, CA USA

On November 17, 2025 Paul Ekman died peacefully in his home in San Francisco. He lived to the age of 91, and his life force and indomitable spirit remained strong right up to the end. For his family, friends, colleagues, and the many people from all walks of life whom he touched, the loss is immeasurable.

Paul was extraordinarily influential in establishing modern affective science. Early in his career, he conducted seminal studies of cross-cultural consistencies and differences in the expression, recognition, and regulation of emotion. This work included a series of remarkable studies with communities in New Guinea that were visually isolated from the Western world. He later developed the Facial Action Coding System (FACS), which enabled objective characterization of observable facial actions. Exemplifying his full-bore scientific commitment, Paul and his collaborator Wallace Friesen electrically stimulated their own facial muscles to map the resultant appearance changes. FACS unleashed an explosion of new research on the role of the face in emotion and has been critical for the development of facial animation and automated facial coding. Paul’s later scientific work included series of laboratory and field studies we conducted together exploring relationships among facial expression, affect, and autonomic nervous system (ANS) activity in emotion, as well as pioneering studies of brain activity and emotion conducted with Richard Davidson. Paul also produced a significant corpus of research on facial and other signs of deceit. This work was enormously influential, spawning individual differences research on lie detection “wizards”, collaborations and consultations with numerous law enforcement and national security agencies, a highly successful book (“Telling Lies”), and even a television show (“Lie to Me”).

In 2000, at a time when his direct involvement in empirical research was lessening, Paul attended a conference in Dharamsala, India organized to introduce Western scientists to His Holiness, the Dalai Lama. Paul was deeply moved by the experience and the two men, both of similar age, began a friendship and productive collaboration. Their efforts led to a new body of work that included public dialogues, books, articles, and web-based materials designed to integrate science with Buddhist practices and to promote compassion and empathy. Paul derived great joy from this unexpected career turn, which also enabled him to work closely with his daughter, Eve Ekman, on the web-based Atlas of Emotions and the Cultivating Emotional Balance training program. Paul felt that these experiences produced important changes in his own emotional life, helping him come to terms with old demons and reducing the tyranny of his negative emotions. As he entered his final years it became clear how much these experiences had transformed him, and how helpful they were in allowing him to derive meaning from and accept the profound changes he was going through.

Despite spending his career in a medical school, where he did not have a readily accessible path to teaching classes and working with graduate students, Paul was deeply committed to training. He pursued this by opening his laboratory to many postdoctoral and visiting scholars; organizing conferences and speaker series; and engaging in thoughtful and supportive exchanges with young researchers around the world. I was one of the many who benefitted from this intellectual generosity, traveling to his laboratory at the University of California, San Francisco in 1981 for my first sabbatical. I did not know it at the time, but over an almost 45-year period we would become collaborators in science and training, companions in adventures small and large, and close friends who were there for each other through life’s triumphs and tragedies. Knowing Paul was transformative for me in ways that I hope were experienced by many others. When I arrived on the doorstep of his Human Interaction Laboratory I was a newly minted tenured associate professor, but in truth I was feeling somewhat disillusioned with my career choice. At times it seemed that academic life put undue emphasis on cleverness, contrivance, careerism, and convenience, and often settled for what was “good enough.” In Paul I found a scientist who was motivated by an undiluted sense of curiosity, had a clear penchant for posing meaningful questions that he cared deeply about, and had the courage and unflagging energy to pursue research without compromise. He had an extraordinary ability to stay the course no matter how long and difficult the path, how challenging the obstacles, and how distant and uncertain the rewards. Importantly, he was not a science delegator but always insisted on “being in the room” where the research was planned, the methods developed and tested, and the actual data collected. I experienced this firsthand when we obtained an NIMH grant to determine if one of our prior U.S. findings about the face and autonomic nervous system would replicate among the Minangkabau of West Sumatra. Despite the considerable hardships, challenges, and life disruptions involved, Paul never considered taking the easy path by sending a team of research proxies. He felt strongly that we had to be there and do the heavy lifting ourselves – and so it was.

There was something invigorating and inspiring in this purity of purpose. Paul reminded me of why I was originally drawn to science, the unique opportunities an academic life could provide, and the importance of asking and trying to answer research questions that I really cared about. His dedication also reminded me of the great privilege afforded to those of us who are able to spend our professional lives following our curiosity, attempting to solve scientific puzzles, and traversing the unpredictable pathways to discovery. The experience of working with him flipped a switch in me and put me on a very different path as a scientist, and as a mentor of future scientists. I, the people I trained, and countless others he interacted with and inspired over the decades, truly bear the indelible Paul Ekman mark. And we all wear it proudly.

Paul Ekman conducted foundational studies that launched the field of affective science, formulated incisive and testable theories, and developed new methodological tools that are widely used, both in scientific research and in countless real-world applications. Consistent with his deep commitment to integrative science, he synthesized and critiqued our field’s most important areas of inquiry, built bridges to other disciplines and the creative arts, and applied affective science to promote compassion and the greater good. At numerous junctures throughout his long career, he engaged in the field’s most spirited debates. While these were sometimes fierce, they helped refine our theories, sharpen our research questions, and establish an intellectual blueprint for the future. As we contemplate his passing, there is solace to be had in savoring the many memories he leaves behind; the new ways he inspired us to see the world; the incredible body of research, theory and methodology he created; his abiding belief that affective science can and should be used to advance the greater good; and the inspiration he provided for so many to pursue affective science with care, rigor, integrity, and passion. In all these ways he will continue to be part of our lives, and the lives of those who come after us.

